# Climatic niche of *Selinum alatum* (Apiaceae, Selineae), a new invasive plant species in Central Europe and its alterations according to the climate change scenarios: Are the European mountains threatened by invasion?

**DOI:** 10.1371/journal.pone.0182793

**Published:** 2017-08-14

**Authors:** Kamil Konowalik, Małgorzata Proćków, Jarosław Proćków

**Affiliations:** 1 Department of Plant Biology, Institute of Biology, Wrocław University of Environmental and Life Sciences, Wrocław, Poland; 2 Museum of Natural History, University of Wrocław, Wrocław, Poland; National Cheng Kung University, TAIWAN

## Abstract

In recent years, a few established populations of *Selinum alatum* have been found in the Eastern Carpathians outside its native range that is the Caucasus and the Armenian Highlands. The species is spreading predominantly in Poland where it can outcompete native plants in certain cases. This study addresses a potential climatic niche of the plant with the special aims to illuminate future spreading and indicate areas suitable for invasion. Our results show that the extent of the favourable habitat of the species is broader than currently known. This suggests that the plant has the ability to become a potential new element in some semi-natural or disturbed ecosystems associated with mountainous areas, especially in Central and Southern Europe. Future (2070) models mostly rendered similar suitability maps, but showed slight differences over particular areas and a contraction of suitable habitats, mainly in the northern part of the non-native range.

## Introduction

Invasion is considered as the second most important threat to biodiversity after habitat destruction [1]. Quest for the ecological mechanism that lies behind the success of invasive species over native species has drawn an attention of research worldwide [2],[3],[4]. Many publications considered key issues in plant invasion ecology and significantly improved our understanding of many aspects of invasions. Different concepts, hypotheses and theories concerning plant invasion ecology were for example reviewed by Richardson and Pyšek [5], and Rai [6]. These authors in detail discuss at least 16 different ecological attributes and theories. The most well known and best studied are two ecologically interrelated hypotheses, i.e., enemy release and fluctuating resources which emphasize that invading species must have access to available resources, e.g., light, nutrients, and water, and that an invading species will be more successful at invading a community if it does not encounter intense competition for these resources from resident species [5]. An extremely important factor, required initially for invasion success, is propagule pressure [6],[7]. Residence time integrates aspects of propagule pressure: the longer the species is present in the region, the greater the size of the propagule bank, and the greater the probability of dispersal, establishment, and the founding of new populations [5]. Further, phenotypic plasticity and/or rapid evolutionary change (caused by genetic drift and inbreeding in founder populations, by intra- and interspecific hybridization in the introduced range creating novel genotypes, and by drastic changes in selection regimes imposed by novel environments) are two not mutually exclusive basic options that are available for an introduced plant species to invade a new region. Either the plant must possess sufficiently high levels of physiological tolerance and plasticity, or it must undergo genetic differentiation to achieve required levels of fitness [5]. More other hypotheses pertaining to success of invasive plant species may also include novel weapon, empty niche, evolution of increased competitive ability or seed plant invasiveness [5],[6].

In Europe almost two thirds (62.8%) of the established plant species were introduced intentionally for ornamental, horticultural, or agricultural purposes. The remaining species were introduced unintentionally, mostly associated with transport vectors, or as contaminants of seeds and other commodities [8].

Probably two of the most economically and ecologically problematic invasive plants in Central Europe are *Reynoutria japonica* Houtt. (Polygonaceae) and *Solidago* sp. (Asteraceae). *Reynoutria japonica* is well known for its high strength of shoot growth sprouting from rhizomes in the spring. Rhizomes can grow up to 2 m in depth, and shoots outgrow areas paved with cobbles and even covered with asphalt. Due to difficult and costly procedures in removing the rhizomes, e.g., in the UK a land for sale on which the knotweed occurs, is much cheaper than the land without these plants [9].

A serious threat to the meadow habitats, especially on areas where mowing stopped, are *Solidago canadensis* L. and *S*. *gigantea* Aiton. These species cause a drastic decrease in diversity of meadow phytocoenoses, but as species of melliferous properties, they have sprung up in some localities after the seeding by beekeepers or as ornamental plants in home gardens. Their presence can be seen as positive in the period of flowering, i.e., especially at the end of the growing season (August/September in Central Europe) when they produce a large amount of pollen and nectar. However, this positive effect refers mainly to honeybees, which arrive in such areas even from remote locations (e.g., a few kilometers).

With respect to other insects directly linked to the areas gradually overtaken by goldenrods, the final effect is definitely unfavourable. For example in Poland, a significant reduction in the diversity of native meadow plants, caused mainly by strong allelopathy of goldenrods, affected the overall wealth of many insect groups [10], and nesting birds [11]. Strong allelopathy of goldenrod along with seeds equipped with pappus that is adjusted for anemochory make these plants dominating in many kinds of plant communities [9].

A species that also produces very big amount of seeds and in Poland is already classified as an invasive plant is *Selinum alatum* (M. Bieb.) Poir. (= *Cnidiocarpa alata* (M. Bieb.) Pimenov & Kljuiykov) (Apiaceae, Selineae). It is native to the Caucasus region and the Armenian Highlands, but its several populations were found recently in Poland [12], about 1,100 km in a straight line from the nearest native locality [13]. Moreover, it seems that the species is naturalised, and colonises new areas and extends its range predominantly within the Bieszczady Mts in the Eastern Carpathians [13].

In its native range, it grows in wet places such as damp meadows, streamsides, flushes and bushy slopes between 1600 m and 2800 m a.s.l. [14]. In Poland, it is mostly found in patches of ruderal communities; accompanying species are then often common meadow species. The favourable conditions to develop are greatly disturbed and therefore open to colonizers. Moreover, *S*. *alatum* was recorded in semi-natural over-fertilised and disturbed fresh meadows, and single specimens were observed within slightly wetter meadows. Additionally, the species occurs at the edges of scrubs and on escarpments of stream banks, as well as near buildings, roads, and railway tracks [12]. Due to latitudinal differences, it is also found in much lower altitudes ranging from about 480 m to 690 m a.s.l.

A few invasive populations established so far occupy the area of about 600 km^2^ [12],[13]. In Polish mountains *S*. *alatum* flowers in July-August [12],[13] and ripe fruits are formed starting since August. The fruit is a schizocarp that, when mature, splits up into two mericarps. It is glabrous, ovate, 3–5 mm long, with 5 distinct wings [12]. The plant grows in clumps and a single umbel can produce even 1,500 seeds in a given season (and one plant forms many umbels, usually not less than a few). The seeds are dispersed by wind. Moreover, the plant shows a tendency for fast, vegetative, expanding growth [12],[13].

In this study, the potential climatic niche of the species is assessed, which may serve as a proxy for the range of the species and predict its expansion. Moreover, based on the current climatic niche requirements, whether any shift in the potential range of the species will occur is tested taking into account future climate change.

## Materials and methods

### Localities

The occurrence data were obtained from personal observations, herbarium specimens, and the literature. The georeferencing process followed that reported by [15] and the coordinates were assigned as precisely as possible to the location of the collection place. If the coordinates were provided, they were verified. The Google Earth (ver. 7.1.2.2041, Google Inc.) application was used to validate all gathered information. In total, 48 localities (including 11 invasive populations) could be precisely placed on the map. Prior to the analyses, this number was reduced by removing duplicate records within one grid cell, which left 44 localities in the modelling (S1 Table).

### Niche modelling

Nineteen bioclimatic variables in 30 arc s (~1 km^2^) from the worldclim database [16] were clipped using biotic regions where occurrences were found. Biotic regions serve as a reliable estimator for the area that is accessible to the species and it is the most operational way to designate correct M which delimitates dispersal potential of a species [17],[18]. For that purpose, the map of terrestrial ecoregions of the world was used [19]. Temperate biomes were clipped at the Ural Mountains which represents a biogeographical barrier for many plant species. To account for the co-linearity and to select the most important bioclimatic variables, the number of the original WorldClim variables was reduced. For that purpose an R package MaxentVariableSelection [20] was used which utilizes similar procedure as Warren et al. [21] with the aim to choose appropriate beta-multiplier and exclude correlated variables. To achieve that a set of models is created with different settings (varying in the value of beta-multiplier and the number of variables) and their performance is assessed by calculating Akaike Information Criterion (AIC), AIC corrected for small sample sizes (AICc), and Bayesian information criterion (BIC). Following criteria were applied: correlation threshold was set to 0.7, contribution threshold to 5 and beta-multiplier was tested in the range from 1 to 15 using 0.5 steps. Settings with the highest AICc score were chosen for modelling.

Additionally it was tested whether any other factors related to topographic or soil properties may be important for modelling distribution of the species. To account for that three separate analyses were run including: selected soil variables, selected soil and climatic variables, and a joint analysis with all (43) available variables. 18 soil variables relevant to plant growth were downloaded from www.soilgrids.org [22] in 250 m^2^ resolution and were upscaled to match bioclimatic variables. Additionally, six topographic variables based on elevation raster were included. Full list of variables is provided in supporting information (S2 Table). Soil and topographic variables were subject to the procedures of variable selection as previously described.

The modelling was conducted using the maximum entropy method implemented in Maxent version 3.3.3k [23] which is widely used and is known for its reliability ([24] and references therein). The maximum iterations were set to 10^4^ and convergence threshold to 10^−5^. For each run, 20% of the data were used and set aside as test points. The “random seed” option which provided random test partition and background subset for each run was applied. The run was performed as a bootstrap with 10^3^ replicates, “auto features” were used, and the output was set to logistic.

Predictions of the future extent of climatic niche were performed using climate projections obtained from Coupled Model Intercomparison Project Phase 5 (CMIPP5) using four “representative concentration pathways” (RCPs: rcp26, rcp45, rcp60, rcp85) which differ in predicted CO_2_ concentration [25]. Only models covering all four representative concentration pathways for the year 2070 (average for 2061–2080) were obtained from www.worldclim.org (S3 Table). To reduce the bias caused by the selection of only one specific model, they were averaged and the ensemble map for each variable in particular RCP was computed. For presentation of the results, continuous grids were converted to binary grids using maximum training sensitivity plus specificity logistic threshold. In the future predictions soil and topographic data were not used due to uncertainties and lack of availability of their future estimates.

To visualize climatic space of the species Principal Component Analysis (PCA) was performed on selected variables, as described in [24].

## Results

### Variable selection

According to MaxentVariableSelection [20], the highest AICc score was assigned to the model with beta-multiplayer = 1. The set of the most important and uncorrelated climatic variables included five from the original 19 and they were: Isothermality (Bio03), Minimum Temperature of Coldest Month (Bio06), Mean Temperature of Wettest Quarter (Bio08), Precipitation of Driest Month (Bio14), Precipitation Seasonality (Bio15). Those variables were distinctive for the observed populations and seem not only to influence the distribution of *S*. *alatum* but also distinguish the Middle Eastern mountain ranges and the Caucasus Mts from their surroundings. Thus, given that this region is the species’ native area it seems that the variables were unambiguous choice. The most important and uncorrelated soil and topographic variables included: roughness, topographic position index and soil organic carbon content. In general they also well characterise mountainous regions.

### Model evaluation

An area under the curve (AUC) for different models ranged from 0.957 to 0.988 which indicates excellent model performances. Additional summary statistics were calculated and involved mean cross-entropy (mxe) which varied from 0.03 to 0.11 and root-mean-squared error (RMSE) that was between 0.09 and 0.17. Both of them reached low scores which indicate a good performance of the model.

### Predictions

**Fig 1:**
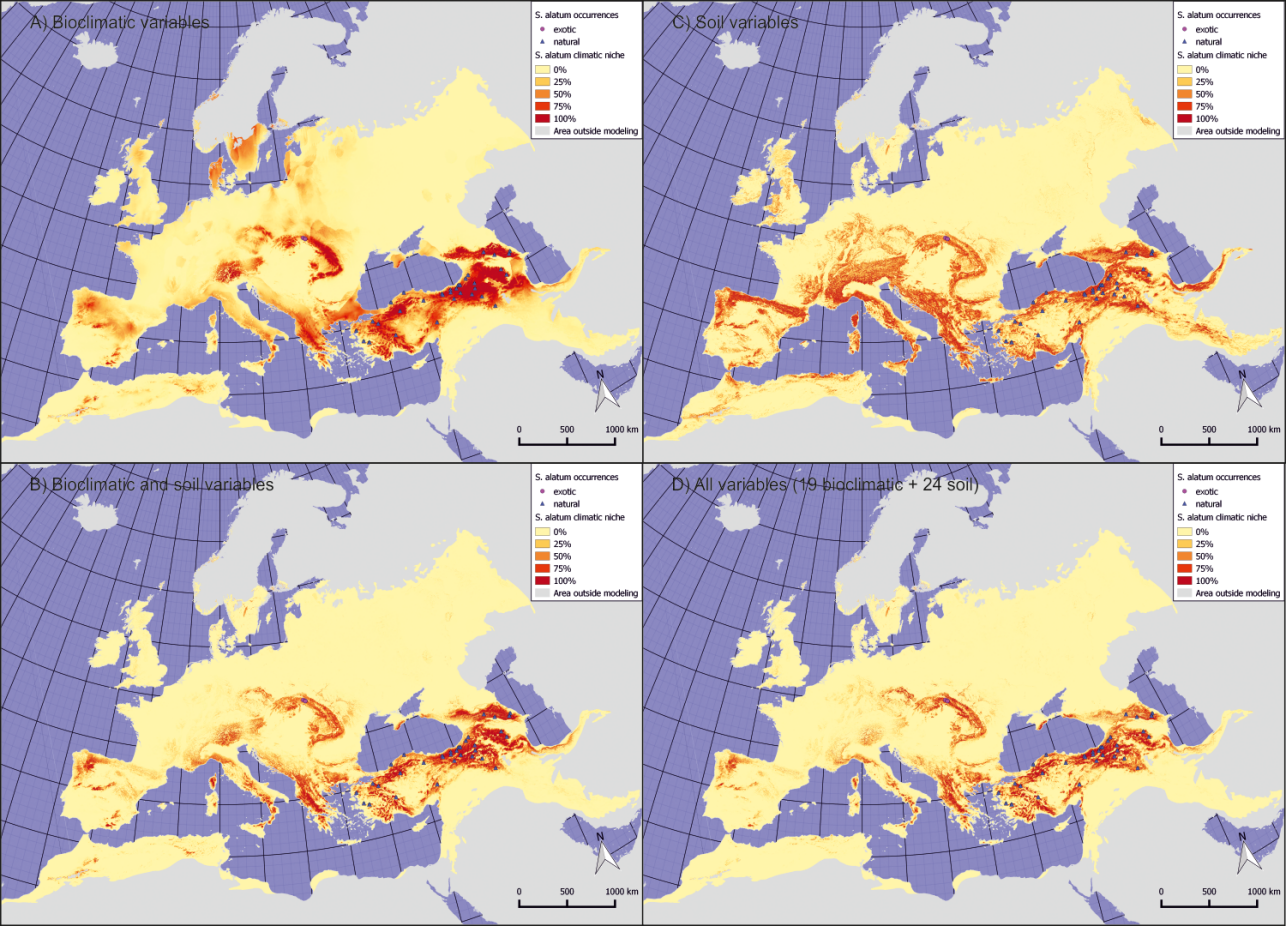
Extent of the climatic niche of *S*. *alatum* in the present time. Darker colours indicate higher habitat suitability. The insets represent modelled potential distribution constructed using following input: A) five selected bioclimatic variables, B) three selected soil and topographic variables, C) five selected bioclimatic variables, and three selected soil/topographic variables, D) all (43) available bioclimatic, soil and topographic variables. The map is projected using Lambert Azimuthal Equal Area (ETRS89/ETRS-LAEA).

According to the model, the most suitable habitats within the native range of the species may be located in the whole Caucasus Mts, the Armenian Highlands, the Taurus Mts, the Pontic Mts, and a mosaic of smaller mountain ranges within the Western Anatolian Plateau that should be considered a western border of the native occurrences (Fig 1). Further suitable habitats found apart from its native range (including also uninhabited areas) comprise the Pindus Mts and adjacent ranges, the Carpathians, the Sudetes, the Eastern Alps, and the Crimean Mts. A few other mountain ranges located in the Mediterranean region and some parts of Scandinavia are also suitable to a lesser extent and mostly are of insular character. Regarding different sets of variables the differences had marginal character and the main area predicted as suitable remains similar. However, some differences exist as for example in the map constructed only with soil variables, vast areas in Western Europe were predicted as suitable (i.e., in Germany and France). Similarly areas in the Apennine and Iberian Peninsulas gained higher suitability. These trends, however, are not visible on predictions which include climatic data suggesting that they may play a role in shaping the western part of the plausible distribution. On the other hand, joint climatic and soil modelling indicated that some areas predicted with the model using only climatic variables were not regarded as suitable. This includes e.g. some areas in Central and northern Europe, central areas of the Carpathian Mts., Corsica, and some smaller areas especially in the Balkan and Apennine Peninsulas.

**Fig 2:**
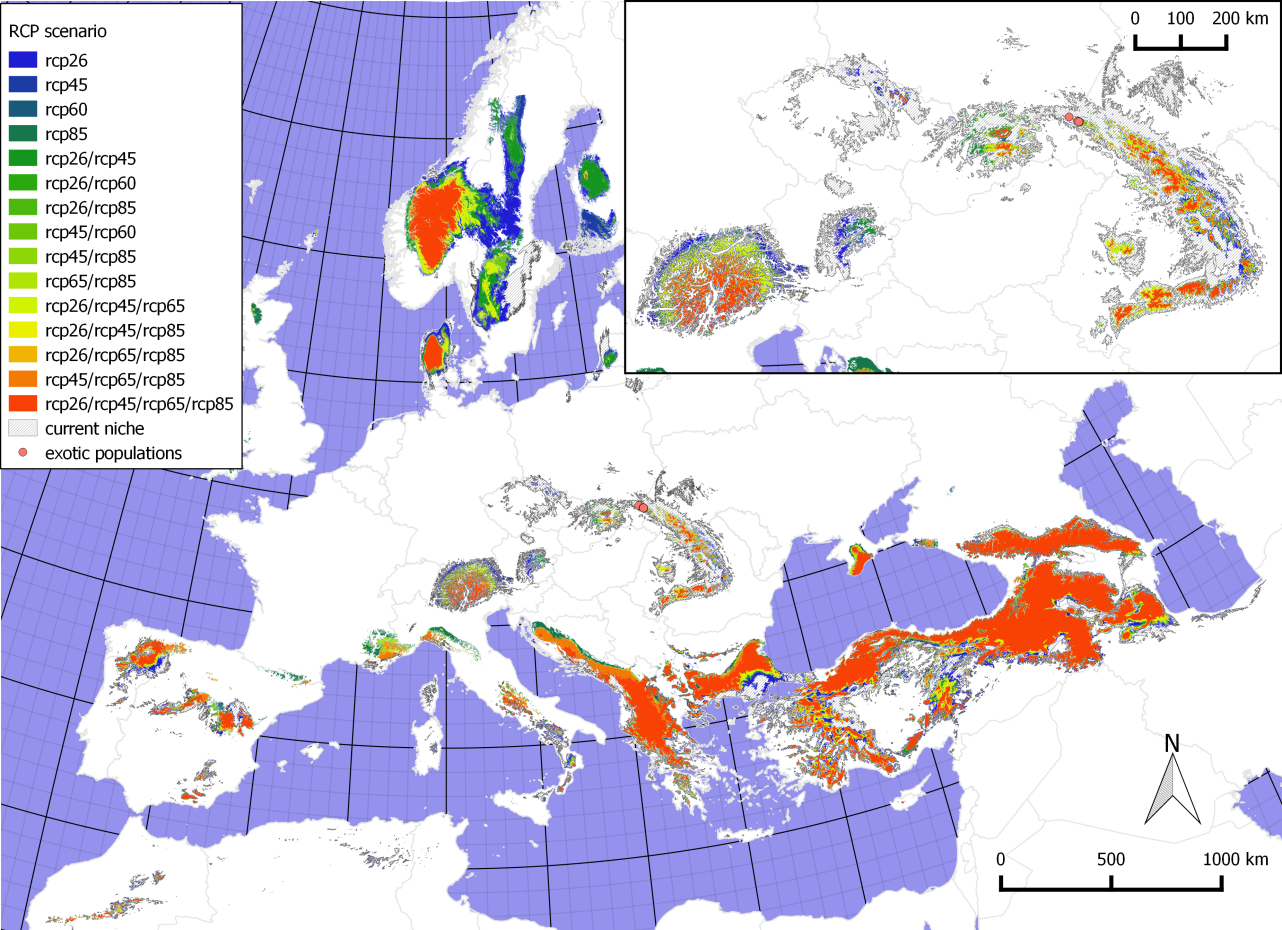
Possible changes in the extent of the climatic niche of *S*. *alatum* in the future (for the year 2070) according to different representative concentration pathways of CO_2_. Extent of the current climatic niche is indicated with a hatched area while different colours depict suitable areas under different scenarios. Inset shows Central Europe where current invasive populations are found–in this region severe shrinkage of suitable habitats may be observed. The map is projected with Lambert Azimuthal Equal Area (ETRS89/ETRS-LAEA).

Four models projected into the future rendered suitability maps with similar general trends but different magnitudes (Fig 2). Irrespective of the model, the native range is the most stable area that changes very little between the future projections and the current climatic conditions. Actually, only in the Anatolian Plateau some southward migrations may be observed and a small-scale contraction of the range within the Caucasus and the Western Anatolian Plateau that corresponds to the upward vertical shift associated with climate warming. Concerning the non-native range, there are larger differences between present and future distributions, as well as among future models. The major range shift differences are observed in the Central European Mountains (i.e., the Carpathians, the Sudetes and the Eastern Alps). In these areas, suitable habitats will decrease and become restricted to the higher altitudes. In scenarios assuming the greater increase of radiative forcing, this change will be more pronounced. The reverse trend is observed in the southern Europe where particular areas are designated as more suitable in scenarios with a higher increase of radiative forcing and include mountain ranges such as the Dinaric Alps, the Maritime Alps, the Apennines, the Eastern Pyrenees, and the ranges in the Iberian Peninsula. Considering non-native ranges, only the Pindus Mts and the Crimean Mts have similar suitable areas in the current and future climates with a slight increase of suitability associated with warmer conditions. In the future scenarios, noticeable new potential areas are indicated in the Rhodopes, the Rila Mts, the Pirin Mts, and the Balkans that in the current climate were supported only averagely. Also a relatively large area in the southern part of the Scandinavian Mts shows high climatic suitability but this region was excluded from the modelling in the current time (as described in the methods).

**Fig 3:**
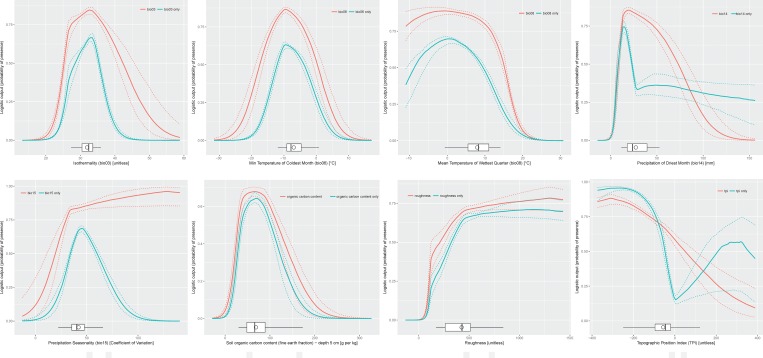
Response curves characterising how each variable affected the Maxent prediction. A red line shows how the logistic prediction changes as each environmental variable is varied keeping all other environmental variables at their average sample value, while a blue line illustrates a Maxent model created using only the corresponding variable. The solid line represents second quantile (median) while dashed lines represent the first and third quantiles. Boxplots show the distribution of values observed in the localities used in the current study. Circle represents mean, horizontal lines minimum and maximum, the box represents first and third quantiles, and vertical line inside delineates median.

The response curves (Fig 3) present the optimal values of the five bioclimatic and three soil/topographic variables. It seems that the most suitable habitat could be characterised by the minimum temperature of the coldest month of around -10°C, maximum mean temperature of the wettest quarter of 15°C, precipitation of the driest month of about 20 mm, non-Atlantic climate and average isothermality. Optimal organic carbon content in the soil should be around 70 g per kg.

**Fig 4:**
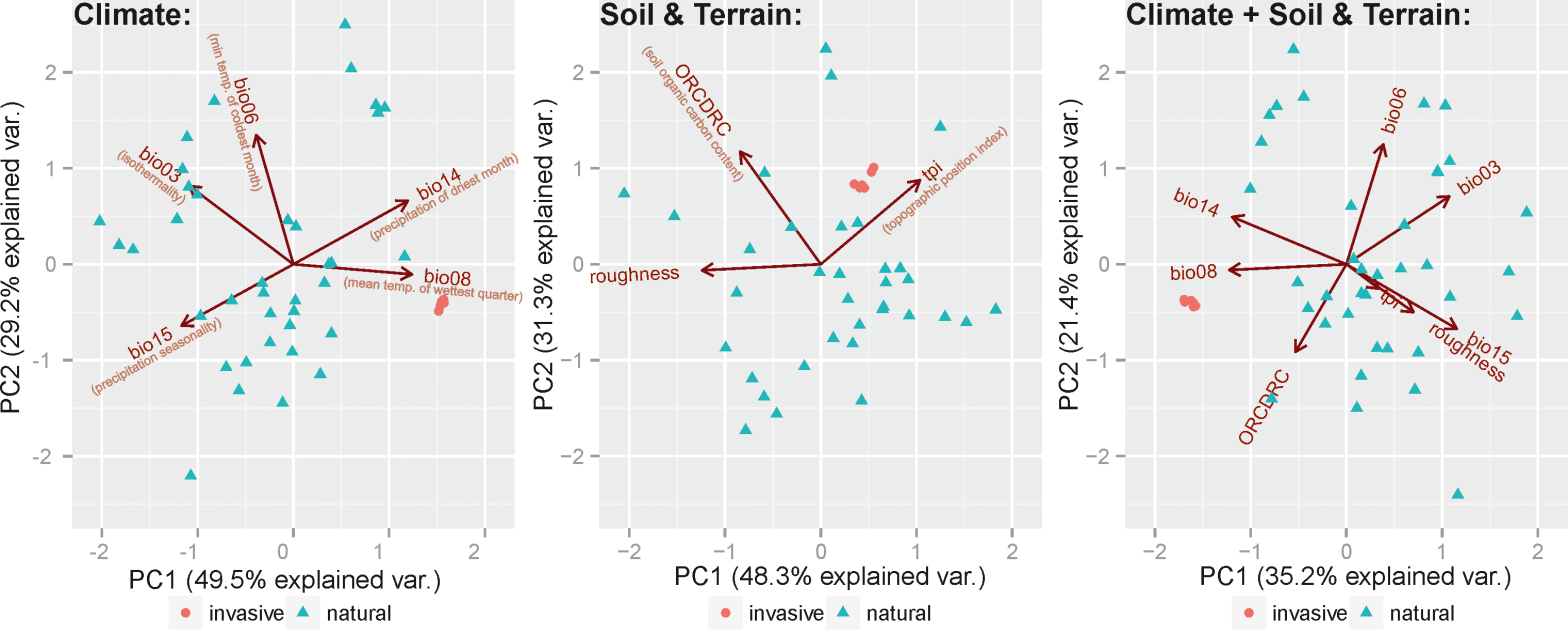
Principal Component Analysis of the variables present in the studied localities of *S*. *alatum*. Cumulatively, PC axes explain 78.7% (climate), 79.6% (soil and terrain), 56.6% (climate, soil and terrain) of the variation present in the dataset. Arrows delineate loadings of variables.

PCA (Fig 4) indicates that in comparison to the native populations, invasive ones tend to occupy a somewhat remote position in the environmental niche; however, this is still within the tolerance of the species. This position seem to be mainly associated with the axis related to mean temperature of wettest month (Bio08) which is higher in exotic locations. Relative to the natural populations, those also exhibit only small variation recorded in the natural ones.

## Discussion

Previous distributional maps of *S*. *alatum* [12],[13] indicate that the western border of the species range is located between the Armenian Highlands and the Anatolian Plateau around a longitude of 40°E. Ecological niche modelling together with occurrences found in the preparation of this study suggest that it extends further west until 27°E. However, this western part of the range in a greater part is represented by patchy appearances, while the continuous range is located in the area that was found in the previous studies.

The potential range of *S*. *alatum* is largely unfilled now; however, further spreading of the species is likely to be observed. If the transport of diaspores occurs, mountainous areas in Europe will mostly be penetrated, mainly the Carpathians (where it is currently found), the Sudetes, the Alps, the Pindus Mts, and the Balkan Mts. A present suitability map notably indicates that favourable habitats are located in the upper parts of Dniester and Prut drainage basins. This may be important in the spreading of the species since rivers are known to aid greatly in dispersal of invasive species [26] and presently known invasive populations of *S*. *alatum* from Bieszczady Mts occur close to the upstream area of those rivers (i.e., only 35 km to the west). To some degree, spreading may also be achieved by an accidental human transportation; for example, mountain tourism.

Irrespective of the variable set potential distribution maps place *S*. *alatum* in the broadly defined mountainous areas. This is also visible in the response curves of the modelling algorithm whose values are associated with this type of environment. Within this habitat some variation exists as it may be seen in the PCA graphs which group locations into few weakly separated assemblages (Fig 4). Obviously exotic sites, which are located only in a narrow area, represent much smaller variation and are placed close to each other. It is likely caused by the founder effect and in the future the plants may follow different scenarios. They will either start to establish populations in new conditions or the slow colonization rate may eventually lead to the shrinkage of the range associated with climate warming. Nevertheless, future spreading of *S*. *alatum* will be restricted, since irrespective of the scenario, the ecological niche modelling indicates that fewer areas will be suitable for colonization. Scenarios with higher concentration of CO_2_ predict a lower coverage in the north and higher in the south of Europe, compared to the scenarios with lower CO_2_ concentrations. Contrary to the non-native range, almost no changes are expected to occur within its native range. Especially, the Dinaric Alps and other mountains in the Balkan Peninsula show different support under varying RCPs where increased CO_2_ levels will make it more suitable. Notably predictions indicate that favourable habitats in the Balkan Peninsula are separated by only 200 km through the Bosphorus strait from the native range of the species. Thus, in this area the species might be treated not as an invasive plant but rather as a natural component that migrates along almost continuous suitable conditions. Following this hypothesis one may also argue that instead of treating this species as invasive it could be considered as a new element in the European Flora that extends its range due to favourable conditions [7],[27].

Species distribution modelling is often used to model areas susceptible to invasive species. As this approach bears clear benefits for the biodiversity conservation and invasive plant management, some caveats may also be encountered. First of all evolutionary processes take place continuously and in many cases it was shown that invasive populations often exhibit novel traits that facilitate their invasions in new areas [28]. These processes have different backgrounds and may involve polyploidy, hybridization, genetic variation, or others [29],[30],[31],[32]. In this case a species may evolve novel adaptations and thus is able to colonize niches different than in its native habitat. Secondly, process of modelling is sensitive to different settings such as, for example: variables used, localities, and modelling method [33]. Here we tried to alleviate this source of errors by using maxent which is one of the most widely used modelling software often outcompeting other methods [e.g., [34],[35],[36],[37]]. Input variables were also screened by computer algorithm for their importance and correlation which makes the choice more objective and not affected by personal preferences. Lastly, to screen for other possible predictions different sets of input variables were used. The last approach revealed that the main area of potential distribution is stable even between as different data sets as climate and soil variables thus adding more support to our analysis.

The Caucasus region with its remarkable biodiversity (it is designated as one of the biodiversity hotspots [38]) is a source of a few invasive plants over the world that include prominent species such as *Heracleum sosnowskyi* Manden., *H*. *mantegazzianum* Sommier & Levier, *Rubus armeniacus* Focke, *Veronica filiformis* Sm. or *Rhaponticum repens* (L.) Hidalgo [39][http://www.iucngisd.org/gisd/]. They were transported either accidentally or on purpose, as in the case of *H*. *sosnowskyi* and *H*. *mantegazzianum*, which were incorrectly considered potentially valuable herbs providing higher yields of forage from meadows [40].

Nobis et al. [12] discuss several possible mechanisms for the introduction of *S*. *alatum* to Poland, including the most probable accidental transportation that took place around the mid-20^th^ century. Taking into account this relatively recent introduction, vast areas of potentially suitable habitats remain to be filled by *S*. *alatum* and the species may become a new component of a few semi-natural or disturbed plant communities in many mountain areas within Europe. The fresh mountain meadows, especially abandoned, over-fertilised and disturbed, as well as escarpments of stream and river banks, also fallow lands, road and railway track margins, and generally the vicinity of human settlements seem to be the most suitable habitats for the species. Moreover, potential habitats are edges of scrubs and patches of slightly wetter meadows [12],[13]. It suggests that in the future the plant may easily strengthen its success to invade all such places. Since a single plant can produce an extremely big amount of seeds, the patches disturbed by *S*. *alatum* rapidly change in terms of both quantitative proportions of indigenous plant species and a species composition.

In sites where this species is naturalised, its presence and spreading may require further monitoring, and interactions with native plants should be studied in more detail.

## Supporting information

S1 Table Lists of localities used in the present study. (DOCX)

S2 Table Full list of variables. (DOCX)

S3 Table Models for year 2070 that were chosen to compute ensemble model for 2070. (DOC)
