# Supplementary material for: Climatic niche of Selinum alatum (Apiaceae, Selineae), a new invasive plant species in Central Europe and its alterations according to the climate change scenarios: Are the European mountains threatened by invasion?
Source: PLoS One. 2017 Aug 14;12(8):e0182793. doi: 10.1371/journal.pone.0182793 (PMC5555634; doi:10.1371/journal.pone.0182793)
Supplement: S1 Table — (DOCX) [file pone.0182793.s001.docx]

**Appendix 1**

**Title**: Climatic niche of *Selinum alatum* (Apiaceae, Selineae), a new invasive plant species in Central Europe and its alterations according to the climate change scenarios: are the European mountains threatened by invasion?

**Authors**: Kamil Konowalik^1*^, Małgorzata Proćków^2^, Jarosław Proćków^1^

^1^Department of Plant Biology, Institute of Biology, Wrocław University of Environmental and Life Sciences, Kożuchowska 5b, 51-631 Wrocław, Poland

^2^Museum of Natural History, University of Wrocław, Sienkiewicza 21, 50-335 Wrocław, Poland

* author for correspondence: e-mail: kamil.konowalik@up.wroc.pl

| **Country** | **Locality** | **Latitude** | **Longitude** |
| --- | --- | --- | --- |
| Georgia | Pirikiti Khevsureti, basin of riv. Arguni, right tributary of the riv. Tergi, near vill. Shatili, the Upper Khonis-Tskali, about 18 km of vill. Shatili, Georgia, 45.23963, 42.57553 , Elevation: 2 121m ± 0m | 42.57553 | 45.23963 |
| Georgia | Lagodekhi Nature Reserve. Along the ridgetop and south-facing slope south of the meteorological station (“Meteo Sta.”) and field cabin, and south of the main trail from Lagodekhi to Meteo Station, from about 41^0^52’13” N, 46^0^21’31”E, southwest along the ridge to a point about 41^0^52’00” N, 46^0^21’00”E, from about 2100-2200 m elevation | 41.87028 | 46.35861 |
| Turkey | Vallee de Djimil [Cimil vadi near İkizdere] (Lazistan) | 40.78874 | 40.58833 |
| Turkey | [B2 Izmir] in Tmolo [= Bozdağ] supra Philadelphiam, *Boissier* | 38.33937 | 28.07632 |
| Turkey | [Bl Manisa] Sypilo [=Sipylus] (Manisa Da.[Dağı]), *Aucher 3605*. | 38.56722 | 27.45472 |
| Turkey | A2(A) Bursa: nr. Broussa (Bursa), Pichler 1874:32! | 40.18333 | 29.05000 |
| Turkey | A3 Zonguldak: 4-5 km E. of Zonguldak, 10 m, *D. 37585!* | 41.45211 | 31.85228 |
| Turkey | A4 Kastamonu: Inebolu, s.l., *D. 21719!* | 41.97158 | 33.76345 |
| Turkey | A5 Amasya: Amasya, *Bornm.* | 40.65018 | 35.83649 |
| Turkey | A7 Giresun: Yavuzkemal, *D. 20747!* | 40.63960 | 38.32114 |
| Turkey | A8 Trabzon: N. slope of Soğanli Da. above Çaykara, 1300 m, *D. 32088!* | 40.57146 | 40.22973 |
| Turkey | Rize: nr. [near] Djimil (Cimil), 1829 m, *Bal.* (type of H. spathyphyllum). | 40.73920 | 40.72408 |
| Turkey | Bl Balikesir: Mt. Ida (Kaz Da.), *Sint. 1883:612!* | 39.70000 | 26.83333 |
| Turkey | B2 Izmir: d. Ödemiş, Boz Da., *Sorger 68-16-121!* | 38.32828 | 28.09987 |
| Turkey | Kütahya: Tahtaköprü forest above Domaniç, 1100 m, *D. 36374!* | 39.83490 | 29.64151 |
| Turkey | B3 Konya: Tekke valley nr. [near] Akşehir, 7 vii 1907, *St. Lager!* | 38.34316 | 31.39713 |
| Turkey | B5. Ankara: Kargasekmez, *T. Baytop 13044!* | 40.42917 | 32.66528 |
| Azerbaijan | Garabagh, pr. Lussogorsk [= Lysogorsk = Lisagor] | 39.68360 | 46.65250 |
| Azerbaijan | distr. Khanlar, around Maral-gel | 40.37838 | 46.31009 |
| Azerbaijan | distr. Kuba, in loco Gil [= Hil] | 41.46477 | 48.33728 |
| Azerbaijan | pr. pag. Leze [Laza] | 41.30056 | 48.11204 |
| Azerbaijan | inter pag. Leze et m-tem Shach-dagh | 41.28670 | 48.08463 |
| Turkey | A7 Giresun: below Tamdere, 1600 m, D. 20628! | 40.50880 | 38.35402 |
| Turkey | Trabzon: Hamsiköy, T. Baytop 14224!, 142461 | 40.68394 | 39.47982 |
| Turkey | Gümüşhane: Tempede (Tenbeda) Sint. 1894: 7537! | 40.45000 | 39.38333 |
| Turkey | A8 Trabzon: N. slope of Soğanli Da. above Çaykara, 1600 m, D. 32100! | 40.56550 | 40.22964 |
| Turkey | Rize: d. Ikizdere, Başköy to Cermanin, Y., 2300 m, D. 21026! | 40.73513 | 40.80822 |
| Turkey | Çoruh: nr. Artvin (Grossheim 7: map 110) | 41.07718 | 41.76299 |
| Turkey | Erzurum: Kop Da. pass, 2440 m, Furse 3743! | 40.03672 | 40.50795 |
| Turkey | Çoruh: nr. Imrehav (Grossheim 7: map 110) | 41.28333 | 42.21667 |
| Turkey | Kars: Sarıkamış, 2100 m, D. 32641! | 40.32998 | 42.58043 |
| Turkey | B6 Maraş: d. Göksun, Binboğa Da. N.E. side of Isik Da. 1900 m, D. 20101! | 38.28104 | 36.59939 |
| Turkey | B7 Erzincan: Sipikor Da., Pirinbaghre [Firin-bahçe], Sint. 1890: 3190! | 39.77119 | 39.50893 |
| Turkey | B8 Erzurum: 20 km from Hinis to Pasinler, 1950 m, D. 46281! | 39.47840 | 41.77044 |
| Turkey | B9 Ağri: 15 km from Eleşkirt to Horasan, E. of Tahir Pass, 2200 m, D. 47188! | 39.86678 | 42.39035 |
| Turkey | Bitlis: Suphan Da., 2800 m, D. 24726! | 38.89064 | 42.79934 |
| Turkey | C9 Hakkari: Cilo Da. above Diz Deresi, 2440 m, D. 23989! | 37.54372 | 43.87332 |
| Poland | Duszatyn (Komańcza district, Sanok county), on the side of a tarmac road - the Kazimierz Sosnowski Trail (the main public hiking trail in the Bieszczady Mountains), 49°18’41.0’’N, 22°7’7.5’’E, alt. 486 masl, 11.07.2013, leg. J. Proćków | 49.31139 | 22.11875 |
| Poland | Strzebowiska - in a meadow, along a gravel road and on abandoned land, alt. 640 masl, 16.07.2007, leg. A. & M. Nobis | 49.18278 | 22.39944 |
| Poland | Strzebowiska - near the tracks of a disusted narrow-gauge railway, alt. 640 masl, 16.07.2007, leg. A. & M. Nobis | 49.18092 | 22.39736 |
| Poland | Strzebowiska - a rubble heap near buildings in the northern part of the village, alt. 620 masl, 18.07.2007, leg. M. Nobis | 49.18628 | 22.40122 |
| Poland | Strzebowiska - roadside escarpment, between the wood and buildings in the southern part of the village (close to a stream), alt. 680 masl, 17.07.2007, leg. M. Nobis | 49.17639 | 22.39650 |
| Poland | Strzebowiska - roadside and slope of a stream bank (near fencing) in the central part of the village, 16.07.2007, leg. A. & M. Nobis, M. Kozak | 49.17847 | 22.39842 |
| Poland | Strzebowiska - roadside and slope of a stream bank (near fencing) in the central part of the village, 16.07.2007, leg. A. & M. Nobis, M. Kozak | 49.17917 | 22.39881 |
| Poland | Strzebowiska - unused meadow on a slope, south of the railway tracks, 17.07.2007, leg. A. & M. Nobis, M. Kozak | 49.18014 | 22.39167 |
| Poland | Krzywe - side of the main road, alt. 580 masl, 19.07.2007, leg. A. & M. Nobis | 49.20239 | 22.36458 |
| Poland | Krzywe - fresh meadow and scrub edge, alt. 610 masl, 19.07.2007, leg. A. & M. Nobis, M. Kozak | 49.19858 | 22.36161 |
| Poland | Przysłup - an abandoned plot of land near the railway tracks, alt. 670 masl, 21.08.2007, leg. A. & M. Nobis | 49.18364 | 22.38972 |
