# Supplementary material for: Climatic niche of Selinum alatum (Apiaceae, Selineae), a new invasive plant species in Central Europe and its alterations according to the climate change scenarios: Are the European mountains threatened by invasion?
Source: PLoS One. 2017 Aug 14;12(8):e0182793. doi: 10.1371/journal.pone.0182793 (PMC5555634; doi:10.1371/journal.pone.0182793)
Supplement: S2 Table — (DOCX) [file pone.0182793.s002.docx]

**Appendix 2**

**Title**: Climatic niche of *Selinum alatum* (Apiaceae, Selineae), a new invasive plant species in Central Europe and its alterations according to the climate change scenarios: are the European mountains threatened by invasion?

**Authors**: Kamil Konowalik^1*^, Małgorzata Proćków^2^, Jarosław Proćków^1^

^1^Department of Plant Biology, Institute of Biology, Wrocław University of Environmental and Life Sciences, Kożuchowska 5b, 51-631 Wrocław, Poland

^2^Museum of Natural History, University of Wrocław, Sienkiewicza 21, 50-335 Wrocław, Poland

* author for correspondence: e-mail: kamil.konowalik@up.wroc.pl

Variables used by MaxentVariableSelection to choose appropriate set of variables and betamultiplier. Topographic variables marked with “Calculated*” as a source were calculated with “raster” package in R using function “terrain” on altitude dataset. Variables used for modeling are marked with bold.

| Variable | Full name | Source |
| --- | --- | --- |
| Bio 1 | Annual Mean Temperature | Hijmans et al., 2005 |
| Bio 2 | Mean Diurnal Range (Mean of monthly (max temp - min temp)) | Hijmans et al., 2005 |
| **Bio 3** | **Isothermality** | Hijmans et al., 2005 |
| Bio 4 | Temperature Seasonality | Hijmans et al., 2005 |
| Bio 5 | Maximum Temperature of Warmest Month | Hijmans et al., 2005 |
| **Bio 6** | **Minimum Temperature of Coldest Month** | Hijmans et al., 2005 |
| Bio 7 | Temperature Annual Range (BIO5-BIO6) | Hijmans et al., 2005 |
| **Bio 8** | **Mean Temperature of Wettest Quarter** | Hijmans et al., 2005 |
| Bio 9 | Mean Temperature of Driest Quarter | Hijmans et al., 2005 |
| Bio 10 | Mean Temperature of Warmest Quarter | Hijmans et al., 2005 |
| Bio 11 | Mean Temperature of Coldest Quarter | Hijmans et al., 2005 |
| Bio 12 | Annual Precipitation | Hijmans et al., 2005 |
| Bio 13 | Precipitation of Wettest Month | Hijmans et al., 2005 |
| **Bio 14** | **Precipitation of Driest Month** | Hijmans et al., 2005 |
| **Bio 15** | **Precipitation Seasonality** | Hijmans et al., 2005 |
| Bio 16 | Precipitation of Wettest Quarter | Hijmans et al., 2005 |
| Bio 17 | Precipitation of Driest Quarter | Hijmans et al., 2005 |
| Bio 18 | Precipitation of Warmest Quarter | Hijmans et al., 2005 |
| Bio 19 | Precipitation of Coldest Quarter | Hijmans et al., 2005 |
| BDTICM_M_250m_ll | Absolute depth to bedrock (in cm) | Hengl et al., 2014 |
| OCSTHA_M_sd1_250m_ll | Soil organic carbon stock in tonnes per ha 5-15 cm | Hengl et al., 2014 |
| CLYPPT_M_sl1_250m_ll | Clay content (0–2 micro meter) mass fraction in % - 0 cm | Hengl et al., 2014 |
| CLYPPT_M_sl2_250m_ll | Clay content (0–2 micro meter) mass fraction in % - 5 cm | Hengl et al., 2014 |
| CRFVOL_M_sl1_250m_ll | Coarse fragments volumetric in % - 0 cm | Hengl et al., 2014 |
| CRFVOL_M_sl2_250m_ll | Coarse fragments volumetric in % - 5 cm | Hengl et al., 2014 |
| SLTPPT_M_sl1_250m_ll | Silt content (2–50 micro meter) mass fraction in % - 0 cm | Hengl et al., 2014 |
| SLTPPT_M_sl2_250m_ll | Silt content (2–50 micro meter) mass fraction in % - 5 cm | Hengl et al., 2014 |
| SNDPPT_M_sl1_250m_ll | Sand content (50–2000 micro meter) mass fraction in % - 0 cm | Hengl et al., 2014 |
| SNDPPT_M_sl2_250m_ll | Sand content (50–2000 micro meter) mass fraction in % - 5 cm | Hengl et al., 2014 |
| CECSOL_M_sl1_250m_ll | Cation exchange capacity of soil in cmolc/kg - 0 cm | Hengl et al., 2014 |
| CECSOL_M_sl2_250m_ll | Cation exchange capacity of soil in cmolc/kg - 5 cm | Hengl et al., 2014 |
| ORCDRC_M_sl1_250m_ll | Soil organic carbon content (fine earth fraction) in g per kg - 0 cm | Hengl et al., 2014 |
| **ORCDRC_M_sl2_250m_ll** | **Soil organic carbon content (fine earth fraction) in g per kg - 5 cm** | Hengl et al., 2014 |
| PHIHOX_M_sl1_250m_ll | Soil pH x 10 in H2O - 0 cm | Hengl et al., 2014 |
| PHIHOX_M_sl2_250m_ll | Soil pH x 10 in H2O - 5 cm | Hengl et al., 2014 |
| PHIKCL_M_sl1_250m_ll | Soil pH x 10 in KCl - 0 cm | Hengl et al., 2014 |
| PHIKCL_M_sl2_250m_ll | Soil pH x 10 in KCl - 5 cm | Hengl et al., 2014 |
| Aspect | Aspect | calculated* |
| Flowdir | Flow direction | calculated* |
| **Roughness** | **Roughness** | calculated* |
| Slope | Slope | calculated* |
| **TPI** | **Topographic Position Index** | calculated* |
| TRI | Terrain Ruggedness  Index | calculated* |
