# Supplementary material for: Climatic niche of Selinum alatum (Apiaceae, Selineae), a new invasive plant species in Central Europe and its alterations according to the climate change scenarios: Are the European mountains threatened by invasion?
Source: PLoS One. 2017 Aug 14;12(8):e0182793. doi: 10.1371/journal.pone.0182793 (PMC5555634; doi:10.1371/journal.pone.0182793)
Supplement: S3 Table — (DOC) [file pone.0182793.s003.doc]

**Appendix 2**

**Title**: Climatic niche of *Selinum alatum* (Apiaceae, Selineae), a new invasive plant species in Central Europe and its alterations according to the climate change scenarios: are the European mountains threatened by invasion?

**Authors**: Kamil Konowalik^1*^, Małgorzata Proćków^2^, Jarosław Proćków^1^

^1^Department of Plant Biology, Institute of Biology, Wrocław University of Environmental and Life Sciences, Kożuchowska 5b, 51-631 Wrocław, Poland

^2^Museum of Natural History, University of Wrocław, Sienkiewicza 21, 50-335 Wrocław, Poland

* author for correspondence: e-mail: kamil.konowalik@up.wroc.pl

Models for year 2070 that were chosen to compute ensemble model for 2070. Each model covers all four representative concentration pathways - RCPs (rcp26, rcp45, rcp60, rcp85), and each bioclimatic variable of particular RCP was averaged separately.

| No. | Name of the model |
| --- | --- |
| 1 | BCC-CSM1-1 |
| 2 | CCSM4 |
| 3 | GISS-E2-R |
| 4 | HadGEM2-AO |
| 5 | HadGEM2-ES |
| 6 | IPSL-CM5A-LR |
| 7 | MIROC-ESM-CHEM |
| 8 | MIROC-ESM |
| 9 | MIROC5 |
| 10 | MRI-CGCM3 |
| 11 | NorESM1-M |
